# Associations of Serum MOTS-c with Exercise Capacity and Antioxidant Markers in Young Males: A Cross-Sectional Study

**DOI:** 10.3390/antiox15101267

**Published:** 2026-10-01

**Authors:** Ziyi Zhang, Xiaonan Zhang, Chengchen Wang, Yong Zhang, Hai Bo

**Affiliations:** 1 Tianjin Key Laboratory of Exercise Physiology and Sports Medicine, Institute of Exercise and Health, Tianjin University of Sport, Tianjin 300060, China; zhangzy427@tjus.edu.cn (Z.Z.);; 2 Department of Military Training Medicines, Logistics University of Chinese People’s Armed Police Force, Tianjin 300162, China

**Keywords:** MOTS-c, mitochondrial-derived peptide, biomarker, exercise capacity, antioxidant markers, oxidative stress, skeletal muscle

## Abstract

Mitochondrial-derived peptide MOTS-c is an exercise-responsive mitokine, yet its associations with exercise capacity and antioxidant markers remain unclear. This cross-sectional study examined serum MOTS-c across three stratified groups of 30 healthy young males aged 18 to 25 years: elite track and field athletes (*n* = 10), recreationally active individuals (*n* = 10), and sedentary controls (*n* = 10). Measurements included serum MOTS-c, SOD, post-exercise MDA, VO_2_max, forced vital capacity, vertical jump height, handgrip strength, back muscle endurance, and post-fatigue back muscle force. Serum MOTS-c increased progressively across groups (athletes: 507.51 ± 67.42 pg/mL; active: 440.32 ± 32.83 pg/mL; sedentary: 319.31 ± 99.07 pg/mL; *p* < 0.001; Hedges’ g = 2.13 for athlete vs. sedentary comparison). Serum MOTS-c was strongly correlated with absolute VO_2_max (r = 0.892), SOD (r = 0.847), post-exercise MDA (r = −0.910), back muscle endurance (r = 0.725), and post-fatigue back muscle force (r = 0.812) (all *p* < 0.001). In exploratory hierarchical regression, post-exercise MDA remained significantly associated with serum MOTS-c after adjustment for VO_2_max, whereas SOD was not independently associated. Handgrip strength showed no significant correlation with MOTS-c (r = 0.315, *p* = 0.090). These findings suggest that serum MOTS-c is associated with exercise capacity and circulating antioxidant markers in young males, although the cross-sectional design precludes causal inference.

## 1. Introduction

Mitochondria regulate exercise performance through multiple signaling pathways. A unique feature of mitochondria is their possession of a circular genome encoding 13 proteins, alongside short open reading frames that produce mitochondrial-derived peptides, including MOTS-c and humanin [1]. MOTS-c has attracted considerable research attention since its discovery by Lee et al. [2]. This 16-amino-acid peptide is widely expressed in skeletal muscle, heart, liver, brain, and testes, and is classified as a mitochondrial hormone or mitokine.

Lee et al. showed that MOTS-c targets the folate–methionine cycle, leading to AICAR accumulation and AMP-activated protein kinase (AMPK) activation [2]. Skeletal muscle was identified as the primary target, where MOTS-c enhances glucose uptake and fatty acid oxidation. The peptide also exerts systemic metabolic effects through inter-organ signaling, including the regulation of hepatic glucose output [3]. Prior work has demonstrated that MOTS-c can translocate to the nucleus and interact with nuclear factor erythroid 2-related factor 2 (Nrf2) to activate antioxidant gene transcription [4], functioning as a mediator of mitochondrial–nuclear communication [5].

Accumulating evidence has established a close relationship between MOTS-c and physical exercise. Reynolds et al. demonstrated that acute high-intensity cycling increased skeletal muscle MOTS-c protein expression by 11.9-fold and circulating levels by 1.5-fold in healthy young males. Exogenous MOTS-c administration significantly improved running capacity in mice, with treated old mice running twice as far as untreated controls [6]. Hyatt reported that long-term voluntary wheel running increased MOTS-c protein levels up to five-fold in fast-twitch dominant muscles [7]. Exercise-induced MOTS-c expression is mediated through the AMPK/peroxisome proliferator-activated receptor gamma coactivator 1-alpha (PGC-1α) pathway. Yang et al. demonstrated that PGC-1α functions as an upstream transcriptional regulator of MOTS-c, forming a self-amplifying positive feedback loop with continued training [8]. Feng et al. confirmed that endurance training enhances mitochondrial respiration through MOTS-c [9].

Despite these advances, human studies remain limited. Domin et al. reported that serum MOTS-c correlated with lower-body muscle strength but not with maximal oxygen uptake in physically active non-athletes [10]. Von Walden et al. found no significant association between plasma MOTS-c and VO_2_max in recreationally active individuals [11]. In contrast, Feng et al. found a significant positive correlation between serum MOTS-c and VO_2_max when comparing marathon athletes with sedentary controls [9].

These discrepant findings suggest that the MOTS-c and exercise capacity relationship may depend on the range of physical activity levels sampled and the specific dimensions of performance assessed. No prior study has systematically examined MOTS-c levels across clearly stratified physical activity levels using a comprehensive battery of assessments for aerobic endurance, muscle strength, antioxidant status, and fatigue resistance. Exercise training simultaneously induces mitochondrial biogenesis and antioxidant adaptation, and MOTS-c is implicated in both pathways. We hypothesized that serum MOTS-c would exhibit a graded association with multidimensional exercise capacity across physical activity levels, and would also be independently associated with circulating antioxidant markers (SOD, MDA).

## 2. Materials and Methods

### 2.1. Study Design and Participants

This cross-sectional study enrolled 30 healthy male university students aged 18 to 25 years from a single university in northern China. Participants were allocated into three groups (*n* = 10 each): elite athletes (track and field, ≥10 h/week structured training, ≥4 sessions/week), recreationally active individuals (≥2 sessions/week, ≥3 h/week), and sedentary controls (no regular structured activity beyond mandatory physical education classes). Group allocation was based on self-reported physical activity levels and was verified by the observed stepwise gradient in VO_2_max across groups. All participants provided written informed consent. The study was approved by the Institutional Ethics Committee of Tianjin University of Sport (approval number TJUS 2024-058, approved on 12 September 2024) and conducted in accordance with the Declaration of Helsinki. Exclusion criteria included diagnosed cardiovascular disease, infectious diseases or other conditions contraindicating exercise participation, current smoking, use of antioxidant supplements or anti-inflammatory medications within four weeks prior to enrollment, poor adherence, and history of adverse reactions to venipuncture.

### 2.2. Anthropometric and Body Composition Measurements

Body height was measured to the nearest 0.1 cm using a calibrated stadiometer. Body weight and skeletal muscle mass were assessed using a multifrequency bioelectrical impedance analyzer (Inbody770, InBody Co., Ltd., Seoul, Republic of Korea). Participants fasted for at least 4 h, abstained from vigorous exercise for 24 h, and voided their bladder immediately before measurement.

### 2.3. Serum MOTS-c Concentration Measurement

Fasting venous blood samples were collected from the antecubital vein after an overnight fast. Participants refrained from exercise, alcohol, caffeine, and high-fat meals for at least 24 h prior. Whole blood was collected into serum separator tubes, allowed to clot at room temperature for 2 h, and centrifuged at 1000× *g* for 20 min at 4 °C. Serum was aliquoted and stored at −80 °C without repeated freeze–thaw cycles. For all biochemical assays, samples were assayed in duplicate, and samples with duplicate CV > 10% were re-assayed. According to the respective manufacturers, the intra-assay and inter-assay coefficients of variation were <10% and <12%. Serum MOTS-c concentration was determined using a commercial ELISA kit (MyBioSource, San Diego, CA, USA; catalog #MBS9346278) according to the manufacturer’s instructions. Optical density was measured at 450 nm using a multimode microplate reader (TECAN Spark, Tecan Group Ltd., Männedorf, Switzerland). Serum was selected for MOTS-c measurement based on the manufacturer’s validated sample matrix, and fasting resting samples were used to reflect basal circulating levels independent of acute exercise effects. Confirmatory analysis by mass spectrometry, as described by Knoop et al. [12], was not performed due to limited sample volume.

### 2.4. Serum Superoxide Dismutase Activity Measurement

Serum SOD activity primarily reflects the extracellular isoform (SOD3) secreted by vascular endothelial cells and skeletal muscle. To minimize the contribution of erythrocyte-derived SOD, blood samples were collected using standard venipuncture technique, serum was separated following clot formation (2 h at room temperature), and all samples were visually inspected for hemolysis. No samples showed visible hemolysis. Serum SOD activity was determined using a commercial assay kit (Cayman Chemical, Ann Arbor, MI, USA; catalog #706002) based on the WST-1 method, with absorbance measured at 450 nm. SOD has been validated as a reliable biomarker of exercise-induced oxidative stress in healthy adults [13].

### 2.5. Post-Exercise Malondialdehyde Measurement

Participants performed an incremental exercise test to exhaustion on a cycle ergometer. The initial workload was 50 watts, with increments of 50 watts every 3 min at a cadence of 60 rpm. This test was performed on a separate day from the treadmill-based VO_2_max measurement, with at least 48 h between tests. Venous blood was collected at 30 min post-exercise, immediately centrifuged at 2000× *g* for 10 min at 4 °C, and plasma was assayed on the same day. Plasma was selected for MDA measurement in accordance with standard practice for thiobarbituric acid-based assays of exercise-induced lipid peroxidation. Post-exercise plasma malondialdehyde (MDA) concentration was measured using a commercial thiobarbituric acid assay kit (Cayman Chemical, Ann Arbor, MI, USA; catalog #10009055), with absorbance read at 532 nm. The 30 min post-exercise sampling time was selected to capture peak exercise-induced lipid peroxidation, consistent with prior studies demonstrating that MDA concentrations reach maximal levels within 30 to 60 min following acute exercise [14]. MOTS-c was measured at rest, whereas MDA was measured 30 min after exhaustive exercise. The two markers therefore reflect different physiological states, and this temporal and matrix difference should be considered when interpreting their association. Post-exercise MDA served as an index of acute exercise-induced oxidative stress. MDA has been established as a validated marker of exercise-induced lipid peroxidation [15].

### 2.6. Maximal Oxygen Uptake and Forced Vital Capacity Measurement

VO_2_max was determined using a cardiopulmonary exercise testing system with breath-by-breath gas analysis. Participants were fitted with a face mask connected to a turbine flow sensor and rested quietly for 5 min before testing. The incremental treadmill protocol commenced at 10 km/h with speed increasing by 1 km/h every 2 min at an initial grade of 1%. Test termination required at least three of the following: oxygen uptake plateau, respiratory exchange ratio exceeding 1.1, heart rate reaching age-predicted maximal heart rate (220−age), or volitional exhaustion. VO_2_max was recorded as the highest 30 s average. Forced vital capacity was assessed using a spirometer prior to the exercise test.

### 2.7. Muscle Strength and Power Measurements

Handgrip strength was measured using a hand dynamometer. All participants were right-hand dominant, and the right hand was tested. Participants stood with arms straight and slightly away from the body, squeezing with maximal effort. The higher of two trials was recorded. Vertical jump height was assessed using a jump measurement platform. Participants performed a countermovement jump with maximal effort. The higher of two trials was recorded. The countermovement jump has been validated as a reliable method for assessing lower-body neuromuscular function [16].

### 2.8. Acute Muscle Fatigue Protocol and Post-Fatigue Measurements

An acute muscle fatigue protocol targeting the back musculature was implemented using an electronic back strength dynamometer. Participants performed repeated maximal isometric contractions with a 10 s inter-contraction interval. Fatigue was defined as a greater than 20% decline in maximal isometric contraction force relative to the initial contractions. The total number of contractions performed to reach the fatigue criterion ranged from 8 to 15 across participants. Although the baseline maximal voluntary contraction (MVC) was observed during the protocol to determine the fatigue threshold, it was not retained as a separate variable, and the percentage of force retained after fatigue could not be calculated retrospectively.

Immediately upon completion, post-fatigue back muscle force was measured. Participants stood upright on the dynamometer platform and pulled vertically upward with maximal effort. The higher of two trials was recorded. Given the absence of a retained pre-fatigue baseline, post-fatigue back muscle force reflects absolute force-generating capacity after exhaustive work and should not be interpreted as a direct measure of fatigue resistance.

Back muscle endurance was assessed using the Biering-Sorensen test, which has demonstrated good reliability for assessing isometric trunk muscle endurance in healthy adults [17]. Participants were positioned prone on an examination table with the upper body unsupported beyond the iliac crest and arms crossed over the chest. The test was terminated when the participant could no longer maintain the horizontal position, as indicated by a visible drop of the upper body below the horizontal plane. A curl-up movement pattern assessment was performed to evaluate abdominal muscle coordination and core stability [18]. Curl-up data were collected but were excluded from the primary analyses due to measurement validity concerns. Participants were positioned supine with knees flexed and feet placed on an electronic scale. The difference between the initial scale reading and the reading during the curl-up was calculated and recorded in kg.

### 2.9. Statistical Analysis

All statistical analyses were performed using SPSS version 22.0 (IBM Corporation, Armonk, NY, USA). Descriptive statistics were expressed as mean ± SD. No outliers were excluded, and all analyses were performed on the full sample of 30 participants. A post hoc power analysis was performed for the primary comparison of serum MOTS-c between groups. With the observed effect size (Hedges’ g = 2.13), α = 0.05 (two-tailed), and *n* = 10 per group, the achieved power exceeded 99%. The sample size of 10 per group was therefore adequate to detect the large between-group differences observed in this study. Comparable sample sizes have also been used in prior MOTS-c investigations, including an acute exercise study in 10 healthy young males [6], a comparison of 10 marathon runners with 12 sedentary controls [9], a cross-sectional study of 20 physically active adults [10], and an exercise trial with three groups of 10 participants each [11].

The normality of data distribution was assessed using the Shapiro–Wilk test. In subgroup analyses, several variables showed deviations from normality. Pearson correlation was applied throughout as the primary method because of its greater statistical power and demonstrated robustness to moderate violations of normality when sample sizes are balanced across groups [19]. Spearman rank correlation coefficients were also calculated and were concordant in direction and comparable in significance with the parametric results.

Levene’s test for equality of variances indicated heterogeneity of variances for MOTS-c, VO_2_max absolute, vertical jump, back muscle endurance, and handgrip strength (*p* < 0.05). Given the balanced design, standard one-way ANOVA was retained, as ANOVA is robust to non-normality under balanced designs with equal sample sizes. Sensitivity analyses using Welch ANOVA confirmed that the observed group differences were not artifacts of variance heterogeneity. For variables with significant Levene’s test, Games–Howell post hoc test was used to control for inflated Type I error under variance heterogeneity. For all other variables, LSD post hoc test was applied. Effect sizes were calculated using Hedges’ g for all pairwise comparisons. Multiplicity correction was not applied across the 10 measured variables, consistent with the exploratory nature of the study. Findings should be interpreted accordingly.

Pearson correlation analysis examined associations between serum MOTS-c and all exercise capacity indicators within each group and in the total sample. Subgroup correlation analyses serve as post hoc descriptive examinations of pattern consistency and are not intended for statistical inference. For the 10 primary correlation tests in the total sample, the Benjamini–Hochberg false discovery rate correction was applied [20].

Hierarchical multiple regression (Enter method) was used to evaluate the independent contributions of aerobic capacity and antioxidant markers to serum MOTS-c variance. The model quantified the proportion of serum MOTS-c variance explained by aerobic capacity and antioxidant markers. VO_2_max was entered in Block 1 based on the AMPK–PGC-1α–MOTS-c pathway. SOD and post-exercise MDA were entered together in Block 2 to assess the additional variance explained by antioxidant markers, consistent with the Nrf2–antioxidant response element (ARE) signaling axis. Collinearity was assessed using variance inflation factor, with values below 5 considered acceptable. All predictors were retained in the final model regardless of significance. Statistical significance was set at a two-tailed alpha level of 0.05. Exact *p* values are reported except for *p* < 0.001, which are presented as this threshold.

## 3. Results

### 3.1. Participant Characteristics

The baseline characteristics of the three study groups are presented in Table 1. No significant differences were observed among groups with respect to age, height, body weight, or body mass index. Descriptive statistics, ANOVA results, and effect sizes for all measured variables are provided in Table S1. Shapiro–Wilk normality test results for all variables in each group are presented in Table S2.

### 3.2. Group Differences in Serum MOTS-c, Exercise Capacity, and Antioxidant Markers

**Figure 1:**
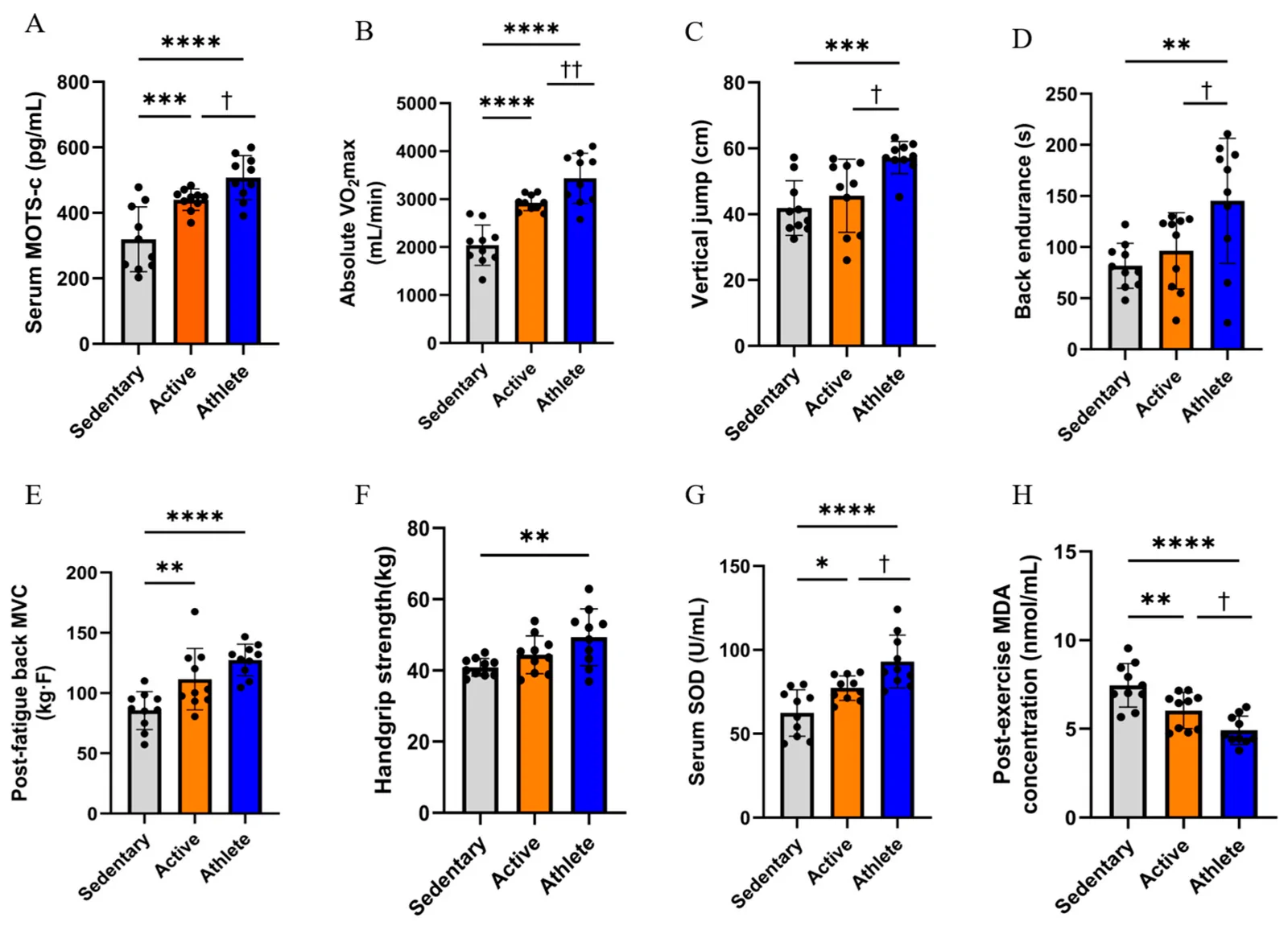
Group comparisons across the three study groups. Panel (**A**) serum MOTS-c concentration. Panel (**B**) maximal oxygen uptake. Panel (**C**) vertical jump height. Panel (**D**) back muscle endurance. Panel (**E**) post-fatigue back muscle force. Panel (**F**) handgrip strength. Panel (**G**) serum SOD activity. Panel (**H**), post-exercise MDA concentration. Each bar represents mean ± SD (*n* = 10 per group). Individual data points are shown alongside each bar. * *p* < 0.05, ** *p* < 0.01, *** *p* < 0.001, **** *p* < 0.0001 vs. sedentary group. † *p* < 0.05, †† *p* < 0.01 vs. active group. Abbreviations: MVC, maximal voluntary contraction; SOD, superoxide dismutase; MDA, malondialdehyde.

Group comparisons for all measured variables are shown in Figure 1. Serum MOTS-c demonstrated a clear stepwise gradient across the three groups, with each pairwise comparison reaching significance. VO_2_max and SOD activity followed the same ascending gradient. Post-exercise MDA displayed an inverse gradient. Vertical jump height and back muscle endurance were significantly greater in the athlete group than in both other groups. Post-fatigue back muscle force was significantly greater in both the athlete and active groups compared with the sedentary group. Handgrip strength was significantly greater in the athlete group than in the sedentary group (Table S1). Skeletal muscle mass was significantly greater in the athlete group compared with both the active and sedentary groups, and was greater in the active group compared with the sedentary group (Table S1). Detailed statistical parameters are provided in Table S1. Welch ANOVA results for variables with significant Levene’s test confirmed that the observed group differences were not artifacts of variance heterogeneity (Table S3).

### 3.3. Correlations Between Serum MOTS-c and Exercise Capacity Indicators

**Figure 2:**
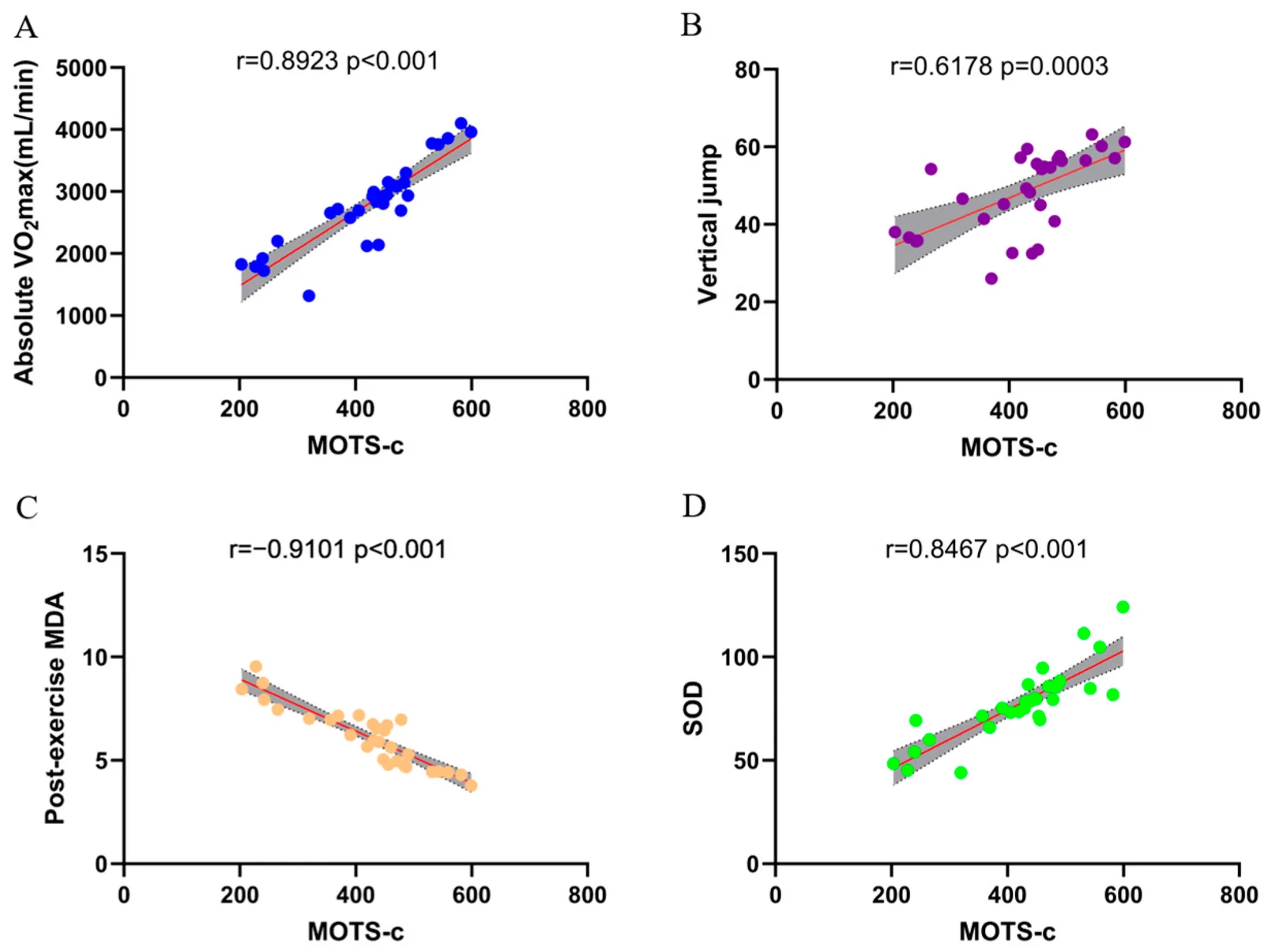
Correlations between serum MOTS-c and key indicators in the total sample (*n* = 30). Panel (**A**) absolute VO_2_max (r = 0.892, *p* < 0.001). Panel (**B**) vertical jump height (r = 0.618, *p* < 0.001). Panel (**C**) SOD activity (r = 0.847, *p* < 0.001). Panel (**D**) post-exercise MDA (r = −0.910, *p* < 0.001). Red line, linear regression fit. Gray band, 95% CI. Abbreviation: VO_2_max, maximal oxygen uptake.

Serum MOTS-c was positively correlated with absolute VO_2_max in the total sample (r = 0.892, 95% CI: 0.787 to 0.947, *p* < 0.001; Figure 2A). Within-group correlations were significant in the athlete and active groups, and a positive trend was observed in the sedentary group (Table S4). For relative VO_2_max, a significant positive correlation was observed only in the athlete group (r = 0.834, *p* = 0.003), though the total sample correlation was significant (r = 0.727, 95% CI: 0.494 to 0.864, *p* < 0.001; Table S4). FVC showed a significant total-sample correlation (r = 0.570, *p* = 0.001), but this association was not significant within any individual group.

Serum MOTS-c was also positively correlated with vertical jump height (r = 0.618, 95% CI: 0.329 to 0.795, *p* < 0.001; Figure 2B), with significant within-group correlations in the athlete and active groups, but not in the sedentary group. Back muscle endurance (r = 0.725, 95% CI: 0.490 to 0.863, *p* < 0.001) and post-fatigue back muscle force (r = 0.812, 95% CI: 0.638 to 0.908, *p* < 0.001) were significantly correlated with MOTS-c across all three groups (Table S4 and Figure S1). Handgrip strength showed no significant correlation with MOTS-c (r = 0.315, *p* = 0.090), with negative or near-zero within-group correlations (Table S4). Skeletal muscle mass was significantly correlated with MOTS-c in the total sample (r = 0.684, *p* < 0.001) but not within subgroups.

### 3.4. Correlations Between Serum MOTS-c and Antioxidant Markers

Serum MOTS-c was positively correlated with SOD activity in the total sample (r = 0.847, 95% CI: 0.708 to 0.924, *p* < 0.001; Figure 2C), with significant within-group correlations in all three groups. A strong negative correlation was observed between serum MOTS-c and post-exercise MDA (r = −0.910, 95% CI: −0.957 to −0.818, *p* < 0.001; Figure 2D), significant across all three groups. Spearman rank correlation coefficients were concordant in direction and comparable in significance with the Pearson results (Table S4). All correlations significant at the nominal level remained significant after Benjamini–Hochberg false discovery rate correction, except handgrip strength (r = 0.315, *p* = 0.090; Table S5). The interrelationships among all measured indicators are summarized in the correlation heatmap (Figure S2).

### 3.5. Independent Predictors of Serum MOTS-c: VO_2_max and Post-Exercise MDA

Partial correlation controlling for absolute VO_2_max revealed that the associations between MOTS-c and antioxidant markers were partially attenuated (Table S6). In the total sample, the partial correlation between MOTS-c and post-exercise MDA controlling for VO_2_max remained significant (partial r = −0.731, *p* < 0.001). After adjusting for VO_2_max, MOTS-c remained significantly correlated with post-exercise MDA in the athlete (partial r = −0.688, *p* = 0.040) and sedentary groups (partial r = −0.823, *p* = 0.006), while the correlation with SOD remained significant in the active group (partial r = 0.697, *p* = 0.037).

Hierarchical multiple regression evaluated the independent contributions of aerobic capacity and antioxidant markers (Table S7). Absolute VO_2_max entered in Block 1 accounted for 79.6% of MOTS-c variance. Addition of SOD and post-exercise MDA in Block 2 explained an additional 10.3% of variance (ΔR^2^ = 0.103, F change = 13.231, df = 2, 26, *p* < 0.001). In the final model, both absolute VO_2_max and post-exercise MDA were independently associated with serum MOTS-c. SOD did not independently predict MOTS-c after accounting for VO_2_max and post-exercise MDA (β = 0.151, *p* = 0.221). The regression results are presented as descriptive aids. With a sample of 30 and three predictors, the model is likely overfit and is not intended as a predictive tool. Complete model parameters are provided in Table S7.

## 4. Discussion

Serum MOTS-c concentrations increased in a graded manner across the three groups and were strongly correlated with both exercise performance and antioxidant markers in young males. The strongest correlations were observed with post-exercise MDA, VO_2_max, and SOD, aligning with the AMPK-PGC-1α positive feedback loop where successive training sessions elevate circulating MOTS-c [6,8]. Lower circulating MOTS-c levels have been reported in obstructive sleep apnea [21], chronic obstructive pulmonary disease [22], type 2 diabetes and obesity [23], polycystic ovary syndrome [24], coronary artery disease [25], and peritoneal dialysis patients [26], as well as in healthy aging men [27]. These observations, together with the present findings, suggest that serum MOTS-c levels vary across populations with distinct physical activity levels and metabolic health status, supporting its potential utility as a biomarker reflecting aspects of systemic antioxidant status, specifically SOD activity and post-exercise lipid peroxidation.

AMPK/PGC-1α signaling has been reported to upregulate both MOTS-c and mitochondrial respiration [6,8,9,28,29], which may provide a molecular basis for the observed co-elevation of MOTS-c and VO_2_max. The discrepancy with studies that found no MOTS-c–VO_2_max correlation [10,11] likely reflects differences in the range of physical activity levels within study populations. Our three-group design captured a broader range of physical activity levels. MOTS-c has also been reported to improve myocardial performance [30] and alleviate diabetic myocardial injury through antioxidant defense [31] in animal models. For the core indicators, within-group associations were concordant in direction with total-sample correlations (Table S4). Correlations with VO_2_max and post-exercise MDA were significant in all three subgroups, indicating that these associations are not solely driven by group-level separation.

Mitochondrial dynamics, particularly the balance between fusion and fission, are critical for maintaining mitochondrial quality and function during exercise adaptation. Acute exercise increases fission events through DRP1 activation, facilitating the removal of damaged mitochondria, while endurance training promotes fusion through MFN1/2 and OPA1, supporting mitochondrial network expansion and respiratory efficiency [28,32,33]. MOTS-c is encoded within the mitochondrial genome, and its secretion may be influenced by changes in mitochondrial morphology and turnover. Whether the elevated circulating MOTS-c observed in trained individuals reflects increased mitochondrial biogenesis, altered fission-fusion dynamics, or enhanced peptide release from a larger mitochondrial pool remains to be determined. Future studies integrating mitochondrial dynamics markers with MOTS-c measurements could clarify this relationship.

Irisin, a myokine cleaved from fibronectin type III domain-containing protein 5 (FNDC5), is released from skeletal muscle during exercise and has been proposed as a mediator of exercise-induced metabolic adaptation [34]. Irisin has been reported to correlate with physical performance and oxidative stress markers in some studies [35,36,37], although the consistency of these associations across studies remains debated. Unlike MOTS-c, which is encoded by mitochondrial DNA and reflects mitochondrial-derived signaling, irisin is a nuclear-encoded myokine. The two peptides may represent distinct but complementary arms of exercise-induced inter-organ communication. Whether MOTS-c and irisin coordinately regulate antioxidant capacity and exercise capacity warrants investigation in future studies that measure both peptides simultaneously.

Serum MOTS-c correlated significantly with vertical jump height, back muscle endurance, and post-fatigue back muscle force. These associations are consistent with reported actions of MOTS-c in skeletal muscle, including casein kinase 2 (CK2)-mediated enhancement of glucose uptake [29], promotion of muscle differentiation [38], reduction in myostatin expression [39], and heat shock factor 1 (HSF1)-mediated preservation of proteostasis [6]. In contrast, handgrip strength showed no significant correlation with MOTS-c (Figure S3), consistent with the absence of grip strength improvement in MOTS-c-treated young mice [9] and the lack of association in COPD patients [22]. Static handgrip strength is more dependent on neural drive and forearm fiber-type composition rather than mitochondrial oxidative capacity, and this selective association may also reflect the differential expression of MOTS-c across fiber types [7]. The greater skeletal muscle mass in athletes compared with sedentary controls may also contribute to the observed MOTS-c gradient, as MOTS-c is predominantly expressed in skeletal muscle.

Three variables displayed a distinctive pattern in which total-sample correlations with MOTS-c differed from within-group associations (Table S4). For handgrip strength, all subgroups showed negative or near-zero correlations, yet the total-sample correlation was weakly positive. This pattern is consistent with Simpson’s paradox. Associations observed in aggregated groups can be reversed or attenuated within individual subgroups. The negative total-sample correlation for curl-up, contrasting with positive within-group trends, likely reflects the measurement method rather than true core stability. Curl-up performance was quantified as the difference between initial and contraction-phase electronic scale readings under the feet. Heavier individuals generate higher baseline readings, producing smaller calculated differences. Athletes had greater mean body weight (75.0 kg) than sedentary controls (70.3 kg), which may explain the paradoxical group ranking. Given these measurement concerns, curl-up data are presented in the Supplementary Materials only (Table S4). Total-sample correlations for FVC and skeletal muscle mass were not supported within subgroups. These observations highlight the value of within-group analyses in stratified designs.

The association between MOTS-c and antioxidant markers is biologically plausible. Prior work has shown that MOTS-c undergoes AMPK-dependent nuclear translocation and interacts with Nrf2 to activate transcription of antioxidant enzymes including SOD [4]. The strong negative correlation with post-exercise MDA is consistent with a potential role for MOTS-c in attenuating oxidative damage, although causality cannot be inferred from these cross-sectional data. Because post-exercise MDA was measured at 30 min after exhaustive exercise rather than at rest, the observed correlations reflect the association of MOTS-c with acute exercise-induced lipid peroxidation, providing a functional index of antioxidant capacity beyond basal oxidative status. These findings are consistent with the Nrf2-ARE signaling axis characterized in cellular studies [5,40,41], which suggests that circulating MOTS-c may serve as a correlate of mitochondrial-driven antioxidant capacity. However, the present study measured only SOD activity and post-exercise MDA, and the TBA method has known limitations, including reactivity of aldehyde derivatives with circulating biomolecules. The observed associations should therefore be interpreted as reflecting a limited aspect of redox biology rather than the full antioxidant system. The partial correlation between MOTS-c and post-exercise MDA remained significant after accounting for VO_2_max (partial r = −0.731, *p* < 0.001; Table S6), indicating that this relationship is not merely a downstream consequence of elevated aerobic fitness.

**Figure 3:**
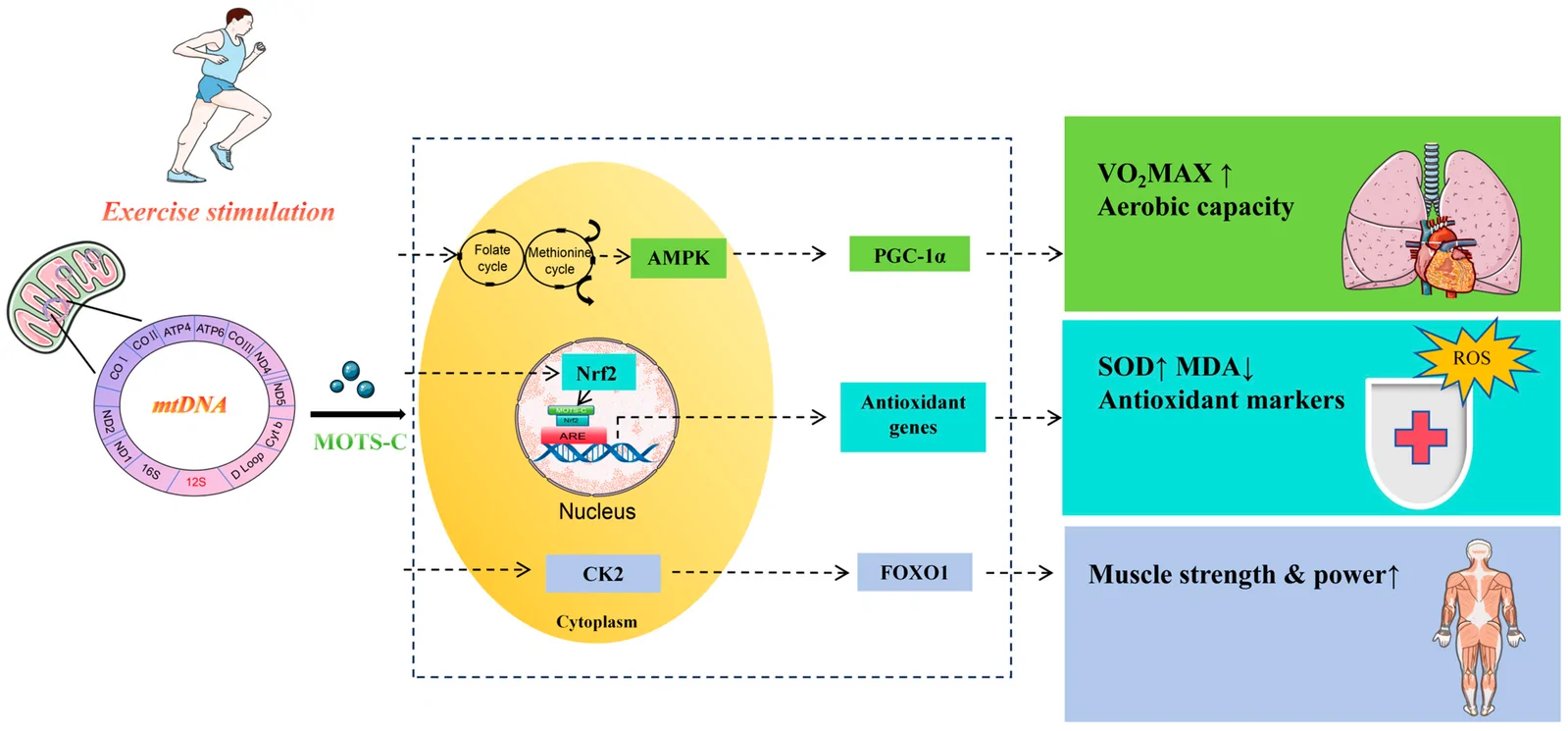
Proposed integrative model of MOTS-c-mediated coordination of exercise capacity and antioxidant markers. The dashed box encloses signaling intermediates and molecular pathways that were not directly measured in the present study, including AMPK, PGC-1α, Nrf2, CK2, and FOXO1. The three outcome domains on the right represent the physiological systems assessed in this study. Arrows denote proposed relationships based on prior mechanistic studies and do not imply established causal relationships.

These findings are consistent with a model in which MOTS-c participates in multiple signaling pathways. The AMPK-PGC-1α pathway is associated with aerobic capacity, the CK2-AKT-FOXO1 pathway with muscle force, and the Nrf2-ARE pathway with antioxidant markers (Figure 3). The signaling cascades in this model are supported by mechanistic evidence [4,6,8,29,38,39], while our cross-sectional associations require causal confirmation.

This study has several limitations. The cross-sectional design precludes causal inference. This study enrolled 30 young Chinese males, and the findings require replication in larger, more diverse cohorts including females, other age groups, and populations with different genetic backgrounds, particularly given the known East Asian-specific K14Q polymorphism [42]. Because participants were sampled from the extremes of the physical activity spectrum rather than from a continuous population distribution, the reported correlation coefficients may overestimate the strength of these associations in a general young male population. The hierarchical regression model, with 30 participants and three predictors, should be interpreted as exploratory. Other redox endpoints, including the GSH/GSSG ratio, GPX and GR activities, protein carbonyls, FAPy glucosidase activity, peroxidases, and hemeprotein levels, were not assessed due to limited sample volume and should be incorporated in future studies. Longitudinal training interventions and studies manipulating MOTS-c availability are required to determine whether the observed associations are causal.

## 5. Conclusions

Serum MOTS-c concentration increased progressively from sedentary to recreationally active to elite athletic groups in young males, and was strongly correlated with multiple dimensions of exercise performance as well as circulating antioxidant markers (SOD and post-exercise MDA). These associations are directionally consistent with, but do not establish, the involvement of the AMPK-Nrf2-ARE signaling axis reported in mechanistic studies. The MOTS-c–post-exercise MDA association remained significant after adjusting for VO_2_max, suggesting an antioxidant link partially independent of aerobic fitness, although causality cannot be established from this cross-sectional design.

## Figures and Tables

**Table 1:** Baseline characteristics of the three study groups.

Variable	Athlete (*n* = 10)	Active (*n* = 10)	Sedentary (*n* = 10)	*p* (ANOVA)
Age (years)	22.3 ± 2.6	21.5 ± 2.9	21.7 ± 2.9	0.812
Height (cm)	178.8 ± 6.9	176.7 ± 6.1	177.3 ± 6.7	0.756
Body weight (kg)	75.0 ± 10.8	75.2 ± 12.2	70.3 ± 12.8	0.543
BMI (kg/m^2^)	23.4 ± 2.5	24.0 ± 2.8	22.3 ± 3.1	0.381

Note. Values are mean ± SD. *p* values from one-way ANOVA.

## Data Availability

The data presented in this study are available on request from the corresponding authors. The data are not publicly available due to participant privacy and institutional ethical restrictions.

## Supplementary Materials

The following supporting information can be downloaded at: https://www.mdpi.com/article/10.3390/antiox15101267/s1, Table S1: Descriptive statistics, ANOVA results, and effect sizes for all measured variables; Table S2: Shapiro-Wilk normality test results; Table S3: Welch ANOVA for variables with significant Levene’s test; Table S4: Pearson and Spearman correlation coefficients between serum MOTS-c and all measured indicators; Table S5: Benjamini–Hochberg FDR correction for Pearson correlations in the total sample (*n* = 30); Table S6: Partial correlation analysis between serum MOTS-c and antioxidant markers controlling for VO_2_max; Table S7: Hierarchical multiple regression predicting serum MOTS-c concentration; Figure S1: Subgroup scatter plots for correlations between serum MOTS-c and back muscle endurance (left panels) and post-fatigue back muscle force (right panels); Figure S2: Correlation heatmap displaying Pearson correlation coefficients among all measured indicators in the total sample (*n* = 30); Figure S3: Non-significant correlation between serum MOTS-c and handgrip strength in the total sample (*n* = 30).

## Abbreviations

The following abbreviations are used in this manuscript:

AMPK	AMP-activated protein kinase
ARE	antioxidant response element
BH	Benjamini–Hochberg
BMI	body mass index
CI	confidence interval
CK2	casein kinase 2
CV	coefficient of variation
ELISA	enzyme-linked immunosorbent assay
FDR	false discovery rate
FVC	forced vital capacity
HSF1	heat shock factor 1
MDA	malondialdehyde
MOTS-c	mitochondrial open reading frame of the 12S rRNA-c
MVC	maximal voluntary contraction
Nrf2	nuclear factor erythroid 2-related factor 2
PGC-1α	peroxisome proliferator-activated receptor gamma coactivator 1-alpha
SOD	superoxide dismutase
VIF	variance inflation factor
VO_2_max	maximal oxygen uptake
WST-1	water-soluble tetrazolium salt 1
