# Supplementary material for: Associations of Serum MOTS-c with Exercise Capacity and Antioxidant Markers in Young Males: A Cross-Sectional Study
Source: Antioxidants (Basel). 2026 Oct 1;15(10):1267. doi: 10.3390/antiox15101267 (PMC13700887; doi:10.3390/antiox15101267)
Supplement: Supplementary file 1 [file antioxidants-15-01267-s001.zip › antioxidants-4510549-supplementary.pdf]

Supplementary Materials

Table S1. Descriptive statistics, ANOVA results, and effect sizes for all measured variables.

Variable	Athlete (n = 10)	Active (n = 10)	Sedentary (n = 10)	p (ANOVA)	Post-hoc	g (A vs S) [95% CI]
Age (years)	22.3 ± 2.6	21.5 ± 2.9	21.7 ± 2.9	0.812	—	—
Height (cm)	178.8 ± 6.9	176.7 ± 6.1	177.3 ± 6.7	0.756	—	—
Body weight (kg)	75.0 ± 10.8	75.2 ± 12.2	70.3 ± 12.8	0.543	—	—
BMI (kg/m²)	23.4 ± 2.5	24.0 ± 2.8	22.3 ± 3.1	0.381	—	—
FVC (mL)	4981.8 ± 664.7	4358.4 ± 533.6	3780.1 ± 580.8	0.001	A > Ac, S	1.85 [0.81, 2.88]
MOTS-c (pg/mL)	507.51 ± 67.42 ***,†††	440.32 ± 32.83 ***	319.31 ± 99.07	<0.001	A > Ac > S	2.13 [1.05, 3.21]
Absolute VO_2max (mL/min)	3436.2 ± 520.9 ***,†††	2924.8 ± 164.1 ***	2039.2 ± 420.4	<0.001	A > Ac > S	2.83 [1.60, 4.06]
Relative VO_2max (mL/min/kg)	43.36 ± 7.56 ***,†	37.43 ± 4.75	32.23 ± 4.98	0.001	A > Ac, S	1.66 [0.66, 2.65]
Vertical jump (cm)	57.19 ± 4.92 ***,†††	45.61 ± 11.08	41.88 ± 8.30	0.001	A > Ac, S	2.15 [1.07, 3.24]

Variable	Athlete (n = 10)	Active (n = 10)	Sedentary (n = 10)	p (ANOVA)	Post-hoc	g (A vs S) [95% CI]
Handgrip (kg)	49.35 ± 7.95 **	44.42 ± 5.30	40.92 ± 2.47	0.010	A > S	1.35 [0.40, 2.31]
Skeletal muscle mass (kg)	41.30 ± 1.59 ***,†††	36.55 ± 1.82 ***	31.12 ± 2.61	<0.001	A > Ac > S	4.51 [2.88, 6.14]
Back muscle endurance (s)	145.22 ± 61.11 **,†	96.33 ± 37.22	81.67 ± 21.96	0.007	A > Ac, S	1.32 [0.37, 2.27]
Post-fatigue back muscle force (kg)	127.43 ± 13.18 ***	111.58 ± 25.43 *	85.50 ± 15.77	<0.001	A, Ac > S	2.76 [1.54, 3.97]
Curl-up (kg)	8.12 ± 0.93 ***,†††	9.85 ± 1.04 **	11.53 ± 1.99	<0.001	A < Ac < S	−2.11 [−3.18, −1.04]
SOD (U/mL)	93.08 ± 15.75 ***,†††	77.30 ± 7.33 ***	62.43 ± 13.82	<0.001	A > Ac > S	1.98 [0.93, 3.03]
Post-exercise MDA (nmol/mL)	4.92 ± 0.80 ***,†††	6.03 ± 1.02 **	7.46 ± 1.22	<0.001	A < Ac < S	−2.36 [−3.48, −1.24]

Note. Values are mean ± SD. p values from one-way ANOVA. For variables with significant Levene's test (MOTS-c, absolute VO_2max, vertical jump, back muscle endurance, handgrip), Games-Howell post-hoc test was applied. For all other variables, LSD post-hoc test was used. Post-hoc p values are uncorrected for multiple comparisons. Multiplicity correction was not applied to post-hoc pairwise comparisons or across the 11 measured variables, consistent with the exploratory nature of the study. * $p < 0.05$, ** $p < 0.01$, *** $p < 0.001$ vs. Sedentary group. † $p < 0.05$, ††† $p < 0.001$ vs. Active group. A, athlete group; Ac, recreationally active group; S, sedentary group; g, Hedges' g; 95% CI, 95% confidence interval for Hedges' g.

Table S2. Shapiro-Wilk normality test results.

Variable	Athlete	Active	Sedentary
MOTS-c	0.879	0.407	0.236
Absolute VO_2max	0.376	0.462	0.678
Relative VO_2max	0.579	0.725	0.004 *
Vertical jump	0.065	0.094	0.126
Back muscle endurance	0.183	0.036 *	0.976
Post-fatigue back muscle force	0.765	0.192	0.852
SOD	0.290	0.462	0.206
Post-exercise MDA	0.358	0.027 *	0.847
FVC	0.019 *	0.521	0.373
Handgrip	0.986	0.805	0.682
Skeletal muscle mass	0.055	0.435	0.842
Curl-up	0.858	0.759	0.506

Note. Values are p values from Shapiro-Wilk test. * $p < 0.05$, significant deviation from normality.

Table S3. Welch ANOVA for variables with significant Levene's test.

**Variable**	**Levene p**	**Welch F**	**df1**	**df2**	**p**	**Standard ANOVA p**
MOTS-c	0.003	11.876	2	15.106	0.001	<0.001
Absolute VO_2max	0.003	24.816	2	14.463	<0.001	<0.001
Vertical jump	0.031	14.066	2	16.163	<0.001	0.001
Back muscle endurance	0.016	4.738	2	15.776	0.024	0.007
Handgrip	0.017	5.990	2	14.879	0.012	0.010

Note. Welch ANOVA was performed as a sensitivity analysis for variables with significant Levene's test ($p < 0.05$, based on mean). Welch p values were concordant with standard ANOVA results for all five variables.

Table S4. Pearson and Spearman correlation coefficients between serum MOTS-c and all measured indicators.

Variable	Athlete (Pearson r)	Athlete (Spearman ϱ)	Active (Pearson	Active (Spearman	Sedentary (Pearson	Sedentary (Spearman	Total r	Total ϱ	95% CI (Total r)
Absolute VO_2max	0.953 ***	—	0.831 **	—	0.585	—	0.892 ***	—	0.787 to 0.947
Relative VO_2max	0.834 **	—	−0.285	—	0.547	0.612	0.727 ***	0.698 ***	0.494 to 0.864
SOD	0.640 *	—	0.634 *	—	0.778 **	—	0.847 ***	—	0.708 to 0.924
Post-exercise MDA	−0.962 ***	−0.976 ***	−0.772 **	−0.891 **	−0.831 **	−0.879 **	−0.910 ***	−0.906 ***	−0.957 to −0.818
Vertical jump	0.689 *	—	0.805 **	—	0.197	—	0.618 ***	0.680 ***	0.329 to 0.795
Back muscle endurance	0.757 *	—	0.747 *	0.636 *	0.902 ***	—	0.725 ***	0.769 ***	0.490 to 0.863
Post-fatigue back muscle	0.771 **	—	0.695 *	—	0.778 **	—	0.812 ***	—	0.638 to 0.908

FVC	0.406	0.200	0.037	—	0.051	—	0.570 ***	0.630 ***	0.270 to 0.770
Handgrip	−0.098	—	−0.493	—	−0.133	—	0.315	0.269	−0.053 to 0.602
Skeletal muscle mass	0.050	—	0.419	—	−0.070	—	0.684 ***	—	0.433 to 0.840
Curl-up	0.405	—	0.400	—	0.252	—	−0.403 *	—	−0.661 to −0.056

Note. r, Pearson correlation coefficient; ϱ, Spearman rank correlation coefficient. Spearman coefficients are reported for variables showing non-normal distribution in at least one subgroup (Table S2). All Spearman coefficients were concordant in direction with the corresponding Pearson coefficients. * $p < 0.05$, ** $p < 0.01$, *** $p < 0.001$. 95% CI, 95% confidence interval for the total sample Pearson correlation coefficient. Given the reduced sample size within subgroups (n = 10 per group), these coefficients should be interpreted as descriptive pattern indicators. 95% CIs for subgroup correlation coefficients are not reported due to the instability of estimates with n = 10 per group. Curl-up data are presented here for completeness but were excluded from the main analysis due to measurement validity concerns (see Discussion).

Table S5. Benjamini-Hochberg FDR correction for Pearson correlations in the total sample (n = 30).

Variable	Raw p	Rank (i)	BH critical value	Significant
Post-exercise MDA	<0.001	1	0.0050	Yes
Absolute VO_2max	<0.001	2	0.0100	Yes
SOD	<0.001	3	0.0150	Yes
Post-fatigue back muscle force	<0.001	4	0.0200	Yes
Back muscle endurance	<0.001	5	0.0250	Yes
Relative VO_2max	<0.001	6	0.0300	Yes
Skeletal muscle mass	<0.001	7	0.0350	Yes
Vertical jump	<0.001	8	0.0400	Yes
FVC	0.001	9	0.0450	Yes
Handgrip	0.090	10	0.0500	No

Note. Significance was determined by comparing each raw p value to its corresponding Benjamini-Hochberg critical value. A raw p value less than its BH critical value was considered statistically significant after FDR correction. Subgroup correlation analyses were not included. Curl-up was excluded from primary analyses and therefore not included in this correction.

Table S6. Partial correlation analysis between serum MOTS-c and antioxidant markers controlling for absolute VO_2max.

Variable pair	Group	Zero-order r	Partial r	df	p
MOTS-c vs SOD	Athlete	0.640	0.399	7	0.288
MOTS-c vs SOD	Active	0.634	0.697	7	0.037
MOTS-c vs SOD	Sedentary	0.778	0.634	7	0.067
MOTS-c vs Post-exercise MDA	Athlete	−0.962	−0.688	7	0.040
MOTS-c vs Post-exercise MDA	Active	−0.772	−0.386	7	0.304
MOTS-c vs Post-exercise MDA	Sedentary	−0.831	−0.823	7	0.006
MOTS-c vs Post-exercise MDA	Total	−0.910	−0.731	27	<0.001

Note. Partial correlations control for absolute VO_2max (mL/min). The sign reversal of the partial correlation in the Active group (zero-order r = −0.772 vs. partial r = -0.386) likely reflects collinearity between absolute VO_2max and post-exercise MDA within this subgroup and should be interpreted with caution given the small subgroup sample size (n = 10).

Table S7. Hierarchical multiple regression predicting serum MOTS-c concentration.

Block	Predictor	B	SE	β	t	p	VIF	R^2	Adjusted R^2	ΔR^2	F change	df	p (F change
1	(Constant)	47.672	36.896	—	1.292	0.207	—	0.796	0.789	—	109.396	1, 28	<0.001
1	Absolute VO_2max	0.134	0.013	0.892	10.459	<0.001	1.00						
2	(Constant)	412.145	104.288	—	3.952	0.001	—	0.899	0.887	0.103	13.231	2, 26	<0.001
2	Relative VO_2max	0.056	0.019	0.371	2.995	0.006	3.96						
2	SOD	0.893	0.712	0.151	1.254	0.221	3.71						
2	Post-exercise MDA	−35.034	8.660	−0.484	−4.045	<0.001	3.68						

Note. Absolute VO_2max, absolute maximal oxygen uptake (mL/min); Relative VO_2max, relative maximal oxygen uptake (mL/min/kg); SOD, superoxide dismutase activity (U/mL); Post-exercise MDA, post-exercise plasma malondialdehyde concentration (nmol/mL). All predictors in Block 2 were entered together as a set (Enter method). With a sample of 30 and three predictors, the model is likely overfit and these results should be interpreted as descriptive.

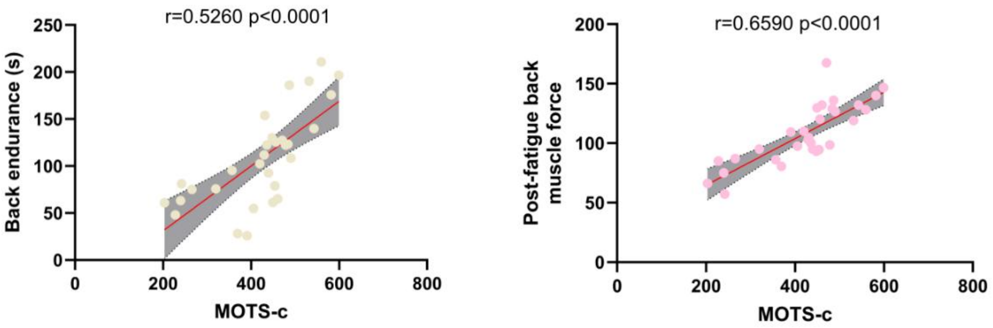

Figure S1. Subgroup scatter plots for correlations between serum MOTS-c and back muscle endurance (left panels) and post-fatigue back muscle force (right panels). Red line, linear regression fit. Gray band, 95% CI. A, athlete group; Ac, recreationally active group; Se, sedentary group.

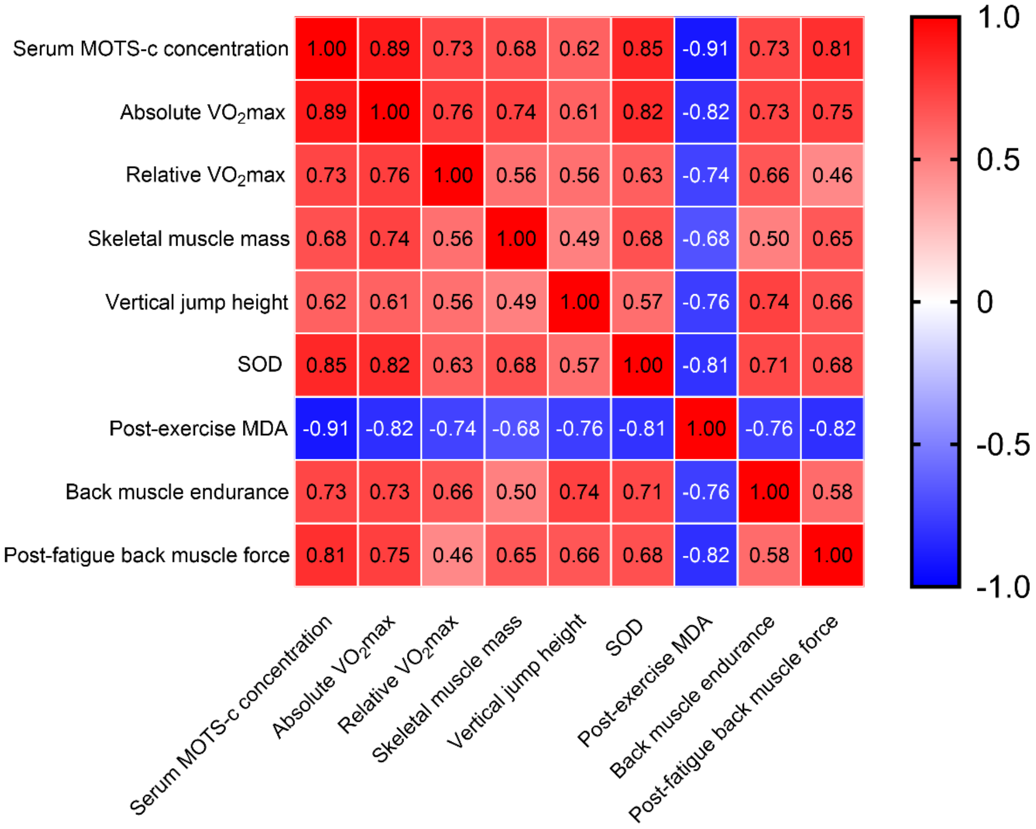

Figure S2. Correlation heatmap displaying Pearson correlation coefficients among all measured indicators in the total sample (n = 30). Warm colors indicate positive correlations and cool colors indicate negative correlations. Color intensity is proportional to correlation strength. MDA, malondialdehyde; SOD, superoxide dismutase.

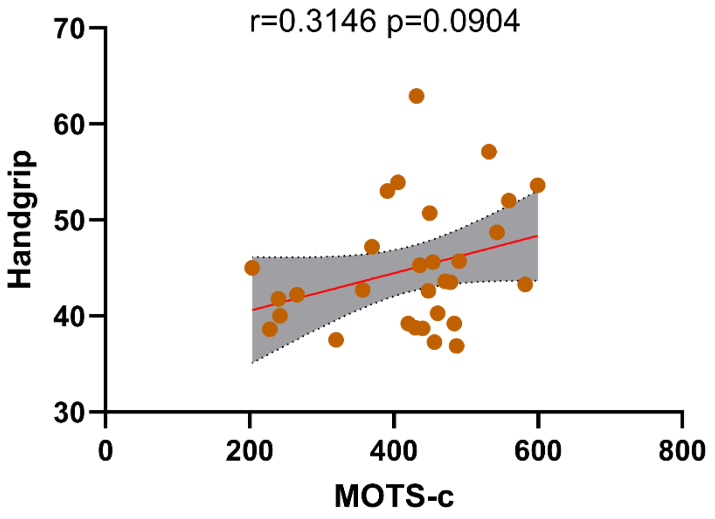

Figure S3. Non-significant correlation between serum MOTS-c and handgrip strength in the total sample (n = 30). Red line, linear regression fit. Gray band, 95% CI.
